# Occurrence of Jaw Osteonecrosis and Frequency of Prophylactic Tooth Extractions Prior to Head and Neck Radiotherapy: A Retrospective Study of 497 Irradiated Patients

**DOI:** 10.3390/jcm14051661

**Published:** 2025-02-28

**Authors:** Inga Krause, Julius Hirsch, Hilke Vorwerk, Boris A. Stuck, Andreas Neff

**Affiliations:** 1 Department of Oral and Craniomaxillofacial Plastic Surgery, UKGM GmbH, University Hospital Marburg, Faculty of Medicine, Philipps-University of Marburg, 35043 Marburg, Germany; hirschju@students.uni-marburg.de (J.H.); neffa@mkg-marburg.de (A.N.); 2 Private Dental Practice, 64285 Darmstadt, Germany; 3 Department of Radiation Therapy and Oncology, UKGM GmbH, University Hospital Marburg, Faculty of Medicine, Philipps-University of Marburg, 35043 Marburg, Germany; vorwerk@med.uni-marburg.de; 4 Department of Otorhinolaryngology, Head and Neck Surgery, University Hospital Marburg, Faculty of Medicine, Philipps-University of Marburg, 35043 Marburg, Germany; sekretariat.hno.mr@uk-gm.de

**Keywords:** humans, oral health, osteoradionecrosis, retrospective studies, risk factors, surgery, oral, tooth extraction

## Abstract

**Background/Objectives:** This retrospective study examined the relationship between prophylactic tooth extraction (PTE) and the occurrence of jaw osteoradionecrosis (JORN) in patients undergoing head and neck radiotherapy (HNR). The primary objective was to determine whether PTE resulted in a JORN rate comparable to that of patients who did not require or undergo PTE. **Methods:** A total of 497 patients were included. The primary predictor variable was PTE, and the primary outcome was JORN occurrence. Statistical analyses included univariate, bivariate, and multivariate regression, as well as Cox regression. The significance threshold was set at *p* ≤ 0.005. **Results:** JORN was more frequent in the PTE group than in patients who did not require or undergo PTE (17.1% vs. 13.0%; hazard ratio [HR] 1.71, 95% CI: 1.08–2.71, *p* = 0.021). However, a significant association could not be confirmed using multiple logistic regression (odds ratio [OR] 1.36, 95% CI: 0.82–2.26, *p* = 0.236). Suggestive associations were observed for HNR dose (HR 1.03 per Gy, *p* = 0.007) and tumor location (pharyngeal HR 0.52, *p* = 0.03; laryngeal HR 0.51, *p* = 0.02). **Conclusions:** Patients with PTE showed a higher JORN rate but the findings were only marginally significant, and no causal relationship was established. The differing results between Cox and logistic regression suggest a time-dependent effect of PTE, with an increased early risk for JORN. Further studies are needed to determine whether greater emphasis should be placed on tooth-preserving measures, limiting extractions before HNR to strictly non-preservable teeth.

## 1. Introduction

Prevention of jaw osteoradionecrosis (JORN) as a serious complication of radiotherapy (RT) for head and neck tumors remains a major challenge in oral and maxillofacial surgery, and huge controversies regarding this issue exist hitherto. Over several decades, prophylactic tooth extraction (PTE) of non-restorable teeth has been alleged to be a common cause of JORN in patients receiving irradiation to the head and neck [1]. Even without PTE, head and neck radiotherapy (HNR) alone causes a hypovascular, hypocellular, and hypoxic state as well as defective fibroblastic activation and regulation within the irradiated tissues. Radiation-induced fibrosis, thrombosis, and obliteration of the vascular system within the bone impairs its capacity to respond to injury and ultimately results in the necrotic process [1,2]. After traumatic events such as TE, it has traditionally been accepted that this necrotic process will be much more critical because of an increased imbalance where cell death and collagen breakdown exceed normal homeostatic levels of cell repair and collagen synthesis, which creates pertinent tissue breakdown and poor healing potential [1,3].

Initially, radical tooth extraction was recommended for all teeth, irrespective of their condition, to prevent JORN [4]. However, this approach was criticized due to overall decreasing JORN rates and improved understanding of its pathogenesis [5,6]. Current strategies emphasize individualized treatment, focusing on dental preservation where possible, and comprehensive pre-RT evaluations to identify at-risk teeth [7].

The recommended minimum time interval between surgical dental treatment and radiotherapy varies in the literature: 7–10 days [8], 2 weeks [9,10], up to 3 weeks [11], or the individually required time until epithelialization of the extraction wound [12,13]. As a standard reference, current guidelines recommend a waiting period of 10 days [7] or 2 weeks [3].

The currently discussed risk factors of JORN include high radiation doses especially a high mandible dose, adjuvant chemotherapy, and tumor location near the mandible. Radiation techniques that limit the radiation exposure of the jaw aim to decrease the risk of JORN [14]. Poor oral hygiene, untreated dental issues, poorly controlled diabetes mellitus, and habits like smoking or alcohol consumption exacerbate the risk [15,16,17]. The mandible is particularly susceptible due to its anatomical and vascular properties compared with the maxilla.

Prophylactic measures pre-RT include detailed dental evaluations with radiographic imaging [18] and management of caries, periodontal disease, and poor oral hygiene [19] Fluoride treatments and motivational oral hygiene counseling are recommended [7]. Post-RT precautions involve avoiding trauma, minimizing denture use during mucositis [2], and performing regular monitoring for complications [20].

Emerging pharmacological approaches, such as pentoxifylline and tocopherol, have shown potential in reducing ORN incidence but require further evidence [21,22,23]. Hyperbaric oxygen therapy remains controversial due to inconsistent results and high costs and limited availability. In addition, the prophylactic use of oral antibiotics as well as the use of platelet-rich plasma and antiseptic mouth rinses in relation with invasive dental procedures have low evidence and limited strength for recommendation [24].

Recent publications including systematic reviews with analysis (SRMA) and several practice guidelines, however, demonstrated a huge controversy regarding the effect of PTE and its timing on the development of JORN [2,25,26,27,28,29,30,31]. The purpose of this study was to answer the following clinical research question: “Does PTE before HNR result in a rate of JORN comparable to the occurrence of JORN as observed in patients not undergoing/requiring PTE?” The null hypothesis (H0) was that no significant difference in JORN development would be found when pre-HNR PTE was versus was not required or performed. Based on clinical reasoning and literature, we expected PTE to exert a protective effect against JORN. The null hypothesis was set conservatively. This reflects the need for an unbiased statistical evaluation as the existing evidence remained inconclusive. It was also assumed that there would be a set composed of ≥1 variables associated with the occurrence of JORN that could be modified to enhance outcomes of patients undergoing HNR. The specific aims of the present study were to (1) conduct a retrospective cohort study on subjects receiving oral health examination prior to and following HNR, (2) estimate and compare the frequency of JORN between subjects with and without pre-HNR PTE of teeth with poor long-term prognosis and/or without the need for PTE, (3) identify associations between PTE (and/or any other potential risk factors) and the presence of JORN and (4) to provide guidance how to design a prospective and/or randomized study to further address the research question.

## 2. Materials and Methods

The presented investigators designed and implemented a retrospective case study. The study sample was derived from the population of head and neck cancer (HNC) patients seen at either the Department of Oral and Craniomaxillofacial Plastic Surgery or the Department of Otorhinolaryngology, Head, and Neck Surgery of UKGM University Hospital Marburg who presented to the Department of Oral and Craniomaxillofacial Plastic Surgery from January 2007 through April 2023 for management of oral health before and after a form of HNR (see below). Patients included in the study were those assessed for dental rehabilitation needs at the Department of Oral and Maxillofacial Surgery at University Hospital Marburg. When indicated, these patients received prophylactic TE to prevent JORN after HNR. Following current clinical guidelines, the determination of PTE necessity included dental status assessment based on radiographic imagining, periodontal condition, and the restorability of teeth. Indications for TE were decay or non-restorable status, periodontal and/or endodontic diseases, and/or uncertain prognosis according to the recommendations of the German guideline for the prevention of JORN [3]. Teeth with uncertain long-term prognosis, advanced marginal periodontitis, and chronic apical periodontitis were extracted.

Rehabilitation was performed either at the hospital or by the patients’ general dentists, with surgical procedures primarily conducted within the clinic. A two-week interval between tooth extraction and HNR was generally maintained as the standard. In some cases, extractions were conducted by the patients’ dentists upon request. A prerequisite for inclusion was radiotherapy for head and neck tumors at the Tumor Center of UKGM, with the radiation field located in the head and neck area. Standard radiotherapy techniques included IMRT, with occasional use of 3D-RT or ion beam therapy at the Marburg Ion Therapy Center (MIT) for specific cases. The treatment plans, radiation doses, and chemotherapy regimens were tailored to the tumor stage and adjusted according to the latest oncological guidelines. In this study, the generally accepted definition of JORN was used, namely, the presence of exposed bone for ≥3 months in a previously irradiated site that is not associated with a persisting or recurrent tumor [1,2,26,27,32].

Institutional review was obtained for this project by the Ethics Committee of Philipps-University Marburg (Approval No. 172/18; date of approval 11 January 2019). The World Medical Association’s Helsinki Declaration and the STROBE guidelines were followed throughout the study. All subjects gave consent for the use of their anonymous data in future research.

### 2.1. Inclusion and Exclusion Criteria

Subjects were selected from the digitalized hospital database and were eligible for study inclusion if they (1) had a histologic diagnosis of a tumor in the head and neck region, (2) were planned for HNR at the Department of Radiation Therapy, and (3) had pre-HNR dental evaluation.

Exclusion criteria included high-dose intake of bisphosphonates or other drugs known to induce bone necrosis before, during, or after tumor therapy. Antibody therapy with Cetuximab (Erbitux^®^) was not an exclusion criterion, as it is not associated with bone necrosis. Patients were also excluded if the follow-up period was shorter than three months or if they did not provide consent for data privacy and the use of their data for research and teaching purposes. Finally, subjects were excluded from the study if medical records were unavailable or incomplete.

### 2.2. Statistical Analysis

The primary predictor variable was pre-HNR PTE, and the primary outcome measure was JORN development. Both parameters were recorded as binary (yes/no). Additional data were collected regarding demographics (age and gender), clinical features (smoking status, alcohol consumption, comorbidity, tumor histology and location, TNM stage, and JORN features [location and interval between TE and diagnosis of JORN]), and therapeutic parameters (HNR modality, dosage, and timing).

After training and standardization of data collection methods, investigators I.K. and J.H. reviewed and abstracted data from the digitalized medical records (ORBIS Information Management System, ORBIS SE, Saarbrücken, Germany) of eligible study subjects using standardized collection sheets (Microsoft^®^ Excel 2016, Microsoft Co., Redmond, WA, USA).

Appropriate uni-, bi-, and multivariate statistics as well as Cox regression analysis were computed for each study variable using IBM SPSS Statistics for Windows, Version 28.0. (IBM Corp., Armonk, NY, USA). Bivariate statistics were performed to estimate the associations between PTE and all other study variables, as well as between the presence of JORN and possible risk/precipitating factors. To develop Cox proportional hazards and multiple logistic regression analyses, variables considered for inclusion into the model were those jointly statistically or near statistically (*p* ≤ 0.15) associated with JORN diagnosis and adjusted as necessary for confounding variables or effect modifiers. Cox regression accounts for time-dependent JORN risk, while logistic regression evaluates the overall PTE–JORN association as binary outcome.

To ensure the robustness of the findings, statistical significance was defined at *p* ≤ 0.005, rather than the conventional *p* ≤ 0.05. This stricter threshold was chosen for several reasons. First, it reduces the risk of false-positive results, which is particularly relevant in retrospective studies where uncontrolled confounding factors may influence outcomes [33]. Second, given the multiple statistical models employed, a stricter significance level mitigates the risk of simulated associations arising from multiple testing. Finally, in a clinical context where study results guide treatment decisions, a more conservative *p*-value threshold ensures that any observed associations are supported by stronger statistical evidence.

Results with 0.005 < *p* ≤ 0.05 were considered suggestively significant and warrant further investigation in prospective studies.

## 3. Results

Descriptive statistics and bivariate associations between study variables and the presence or absence of pre-HNR PTE are presented in Table A1. A total of 729 patients were identified from the hospital registry and evaluated for JORN. After applying the inclusion and exclusion criteria, the final study sample consisted of 497 patients with a mean age of 61 ± 11.0 years (range, 14–91). Among them, 382 (76.9%) were male, and 217 (43.6%) were active smokers. Treatment modalities included IMRT (312 patients, 62.8%), 3D-RT (141 patients, 28.4%), and particle therapy (32 patients, 6.4%).

### 3.1. JORN Incidence and PTE Association

Of the 497 patients, 228 (45.9%) had been apt to pre-HNR removal of teeth due to non-restorable teeth or poor long-term prognosis. JORN was observed in 39 patients (17.1%) in the PTE group compared to 35 patients (13.0%) in the non-PTE group, resulting in an overall JORN incidence of 14.9% (74/497). The median follow-up time was 38 months (range, 3–207 months).

A significantly higher proportion of PTE patients underwent primary HNR (*p* = 0.0004). There was no statistically significant difference in JORN incidence between patients receiving primary (70–75 Gy) versus adjuvant HNR (56 Gy) (*p* = 0.46, regression analysis).

Overall, JORN was more frequently observed in the PTE group (17.1%) compared to the non-PTE group (13.0%), with a relative risk (RR) of 1.38 (95% CI, 0.84–2.26; *p* = 0.209). The number needed to harm (NNH) was 24, indicating that for every 24 patients undergoing PTE, one additional case of JORN may occur. The post hoc power of this comparison was 25.0%, suggesting limited statistical power to detect a significant difference.

### 3.2. Time-Dependent Patterns of JORN Development

Among all JORN cases (*n* = 74), 32 (43.2%) developed JORN within the first year after HNR, and 68 (94.4%) occurred within five years post-HNR. The early incidence was notably higher in the PTE group, where 51.3% of JORN cases occurred within the first year, compared to 32.3% in the non-PTE group. A significant inverse relationship between the time since HNR and JORN occurrence was observed (linear regression: R = −0.762 [95% CI, −3.86 to −0.85], *p* = 0.006). This indicates that JORN risk declines over time.

### 3.3. Regression Analyses and Predictors of JORN

Three variables with suggestive statistical significance in predicting JORN were identified in multivariate analyses:

1. HNR dose (HR = 1.03 per Gy [95% CI, 1.01–1.05], *p* = 0.007; OR = 1.03 [95% CI, 1.00–1.05], *p* = 0.03)
2. Primary tumor site (pharynx) (HR = 0.52 [95% CI, 0.31–0.87], *p* = 0.03; OR = 0.33 [95% CI, 0.13–0.81], *p* = 0.02)
3. Primary tumor site (larynx) (HR = 0.51 [95% CI, 0.14–0.77], *p* = 0.01; OR = 0.51 [95% CI, 0.29–0.90], *p* = 0.02)

Both pharyngeal and laryngeal tumors were associated with a lower risk of JORN when using oral tumor location as the reference.

PTE was identified as suggestively significant in Cox proportional hazards regression (HR = 1.61 [95% CI, 1.01–2.57], *p* = 0.044), indicating a 61% increased risk of JORN in PTE patients. However, this association was not confirmed in the multiple logistic regression model (OR = 1.36 [95% CI, 0.82–2.26], *p* = 0.236), suggesting that the effect may be time-dependent rather than a consistent risk factor.

The term “suggestively significant” is used here to describe findings with *p*-values between 0.005 and 0.05, which may indicate a potential association but do not meet the stricter threshold for definitive statistical significance. While these results suggest that PTE may increase early JORN risk, additional research with higher statistical power and prospective study designs is required to confirm this relationship.

The omnibus test of the multiple regression model (*p* = 0.012) indicated that at least one predictor variable was significantly associated with JORN. Cohen’s d value of 0.06, along with 100% specificity and 0% sensitivity, suggests that the overall model’s explanatory power remains limited. Neither pre-HNR PTE nor chemotherapy were found to be significant independent risk factors for JORN in the final multivariate model.

## 4. Discussion

**Figure 1:**
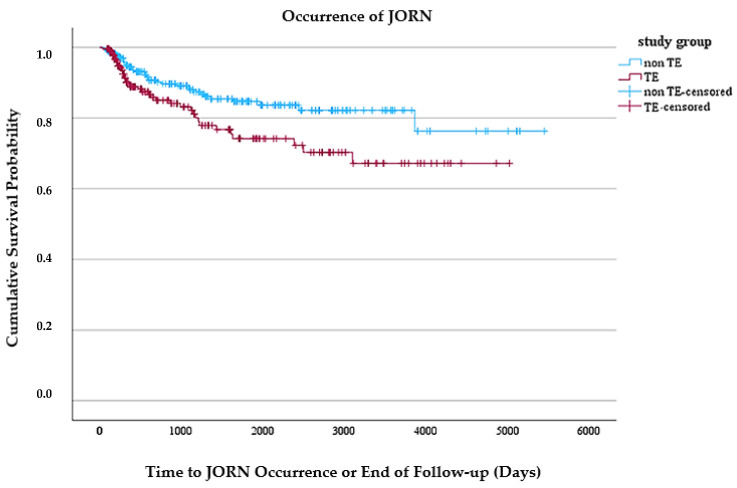
Kaplan–Meier survival analysis for the occurrence of jaw osteoradionecrosis (JORN) in patients with and without prophylactic tooth extraction (PTE). The *y*-axis represents accumulated survival probability, while the *x*-axis shows the latency period until JORN occurrence or the end of follow-up (in days). Here, “non-TE” (blue) represents patients without PTE, while “TE” (red) represents patients who underwent PTE. Censored observations are marked with crosses.

The purpose of this study was to examine the occurrence of JORN in patients with and without pre-HNR PTE. It was hypothesized that PTE would not show significant differences in the frequency of JORN (H0) and probably exerted a protective effect against JORN. The results did not refute the H0, but there are suggestively significant results indicating a potential relationship between PTE and JORN in the Cox regression analysis that are contrary to the H0 hypothesis. However, it is important to emphasize that these findings do not necessarily imply causation. The results showed that PTE patients had a 61% higher risk of suffering JORN as non-PTE patients (Cox regression’s HR, 1.61; 95% CI, 1.01 to 2.57). This should be interpreted with caution as only one in every twenty-five irradiated HNC patients develop JORN associated with pre-HNR PTE (NNH = 25), and two regression analyses yielded different results. The different results of Cox regression and logistic multiple regression can be attributed to a time-dependent effect of PTE on JORN occurrence. While the model assumes a constant hazard ratio over time, the Kaplan–Meier curve (Figure 1) demonstrates a markedly steeper decline in the occurrence of JORN (“survival”) in patients with tooth extractions in the first year and then continues relatively parallel to the control group thereafter. These findings suggest, from a clinical perspective, that PTE may contribute to an increased early risk of JORN rather than a uniform long-term effect. This could be due to post-extraction wound healing challenges in irradiated tissues, emphasizing the need for careful patient selection and optimized pre-HNR dental management. The lack of a significant association in logistic regression also highlights that time-dependent analyses are needed when evaluating treatment effects in JORN development. Future studies should further explore this pattern to refine clinical decision-making regarding PTE in HNR patients.

From a clinical standpoint, these findings suggest that PTE, while intended to reduce the risk of JORN, may instead increase susceptibility in the early post-extraction phase. This aligns with previous studies that highlight the importance of adequate healing time before initiating HNR. Current guidelines recommend a waiting period of 10 days to 2 weeks between tooth extraction and radiotherapy, but whether this is sufficient in all cases remains uncertain. A more individualized approach that considers individual healing capacity, radiation dose planning, and tooth preservation may be more effective in minimizing JORN risk given that the majority of JORN cases in the PTE group occurred within the first year post-HNR (51.3%).

A post hoc power analysis was conducted to evaluate whether the study had sufficient statistical power to detect the observed effect size. Given the effect size Cohen’s h = 0.115, a two-sided significance level of α = 0.005, and a target power of 80%, a total of 4034 patients would have been required (2017 per group) to adequately detect this effect. However, the actual sample size of 497 patients over more than 10 years was substantially lower, resulting in an estimated power of only ~20% for detecting small effects.

This indicates that the study was underpowered to detect minor effects of PTE on JORN, leading to a high probability of false-negative results. However, it was adequately powered to rule out a strong effect of PTE, suggesting that if an association exists, it is likely small and of limited clinical relevance. Given the constraints of a single-center study with long-term follow-up, achieving a sample size exceeding 4000 patients was not realistic. Although the study’s power was insufficient to detect mild effects, its findings provide important insights into the development of JORN.

Recent research evidence documented pre-HNR PTE as a risk factor of JORN [26,28,34,35,36]. The JORN incidences range from 0.55% to 20.8%, with 2.2–100% of cases attributed to the pre-HNR PTE [2,26]. However, other SRMAs and the practice guidelines of the German Association for Oral and Maxillofacial Surgery, the Royal College of Surgeons of England, the British Society for Disability and Oral Health, and the US National Comprehensive Cancer Network still recommend, if possible, a minimal 10-day interval between removal of non-functional or poorly prognosed teeth and HNR (even though data from a Taiwanese nation-wide cohort study [*n* = 24,353] indicated that a 2-week interval between TE and HNR initiation did not significantly reduce the JORN incidence [Kaplan–Meier estimator for JORN risk: log-rank *p* < 0.0001] [30]).

The aims of the prophylactic pre-HNR TE are to reach optimal wound healing and prevent further traumatic events in irradiated oral tissues, which can inevitably increase the risk of JORN [3,26,28,30]. However, from a clinical point of view PTE can have significant consequences for the patient, such as nutritional impairment during treatment [37] and a difficult dental restoration in the future [38]. Extractions in the anterior tooth bearing area can worsen the mental state of the patient as mucosa-supported protheses and removable temporaries cannot be worn during HNR [7]. In addition, PTE delays the start of the HNR and further treatment.

In reviewing the literature, the prevalence of the JORN was reported to have declined from 11.8% before 1968 to 5.4% between 1968 and 1992 and to 3% after 1997 [32].

The relatively high JORN incidence in this study could be linked to the lower hospital volume in Marburg (i.e., 729 HNR per 16.3 years or ~45 cases/year), when compared to other studies, for example, the recent Danish and British studies presenting 1224 and 1118 HNR cases within 9 and 10 years, respectively (or 136 and ~112 patients/year) [28,35]. In contrast, the recent Australian and Brazilian studies revealed JORN incidences of 18.2% and 30.6% from a cohort of ~32.5 and ~5.6 HNR cases/year, respectively [29,36].

In the above cited British study, those authors warned of surgical interventions such as tooth extraction and dental implant placement in the bone exposed to a dose of 55–60 Gy, noting that dental surgery should be avoided when an HNR dose > 60 Gy is used [28]. Recent accumulating evidence confirms this suggestion because almost 100% of JORN develops in patients receiving a total radiation dose ≥ 60 Gy [27,29,30,32]. Also, the multivariate analysis in this study supports the significant relationship between HNR dosage and the occurrence of JORN

On average, in the present study, there is a 3% increase in the odds of developing JORN for every one Gy increase in radiation dose, when other confounders are constant. This OR is less than half of the odds reported by investigators at Memorial Sloan Kettering Cancer Center (MSK) in the US, that is, a 7% increase in the odds of JORN for every one Gy increase, despite the statistically significantly higher HNR dosage (Marburg: 68.7 ± 9.4 Gy vs. MSK: 59.4 ± (estimated)13.4 Gy; unpaired *t*-test: *p* < 0.0001; 95% CI, 7.29 to 9.91) [12]. It can partly be explained that this study included all HNC patients who underwent HNR, while the MSK study analyzed JORN after HNR for oral and oropharyngeal carcinomas, where HNR tends to damage oral tissues more aggressively than in case of other HNCs. As such, 82% of the JORN patients treated at MSK had no prior traumatic event including dentoalveolar procedures. Furthermore, all the patients were treated with IMRT, whereas only 65.2% underwent IMRT in this study, as IMRT in 2007 was just on its way to becoming a standard for HNR in Marburg.

IMRT is more selective regarding the cancer area with a sharp dose falloff; thus, it is expected to diminish the number and the intensity of irradiation side effects, including the JORN incidence to a level of ≤5% [26,32,34,35,36,39].

This study showed no significant reduction in JORN incidence among patients with IMRT in comparison to 3D-RT (15.6% (22/141) to 14.5% (47/324); OR, 1.09 [95% CI, 0.63 to 1.89]; *p* = 0.76). These findings are similar to the results of a Swiss study (10.2% (9/89) to 11% (16/145); OR, 0.92 [95% CI, 0.39 to 2.16]; *p* = 1.0) [38].

Given the limitations of the retrospective study design and the focus on JORN, no specific recommendations regarding the optimal doses for HNR can be derived, as the impact of radiation dose on tumor cure rates must not be overlooked. While a reduction in dose might decrease the risk of side effects, such as osteoradionecrosis, it could also compromise therapeutic success and tumor control. This study did not analyze tumor outcomes and did not utilize randomized data, limiting the ability to establish causal relationships among dose, treatment efficacy, and side effects. Future prospective studies are needed to optimize the balance between effective tumor control and minimizing adverse effects.

Taken together, PTE is an invasive treatment that should therefore have a justified indication. If the results and trends were to be confirmed in a targeted prospective study of HNC patients undergoing IMRT PTE, it may even be considered to remove only symptomatic non-restorable teeth, irrespective of the TE-IMRT sequence. Additionally, this would shift the focus toward tooth-preserving methods as caries therapy, professional dental cleaning, oral hygiene instruction, and fluoridation [19,20]. Therefore, more robust evidence (prospective or randomized studies) is required to clarify whether more emphasis should be placed on these tooth-preserving measures or whether only strictly non-preservable teeth should be removed prior to HNR.

Nevertheless, his study addresses an important clinical issue regarding the relationship between PTE and JORN, which has significant implications for patient outcomes and quality of life. By employing both logistic regression and Cox regression analyses, the study explores the time dependent effects of PTE on JORN incidence, offering information regarding a differentiated understanding of the potential relationship. Furthermore, by comparing and contextualizing the findings with previous research, the study enhances the discussion and provides a foundation for future investigations.

The results of the present study, however, should be viewed within multiple limitations of the design and the data. First, similar to other retrospective cohort studies, this investigation was constructed from a database of healthcare records that have already been collected by different healthcare providers [40]. The inter-observer agreement is not known, and study variables considered as the risk factors for JORN in other reports were not fully identified and subsequently recorded. Therefore, these variables cannot be used in the analysis, e.g., oral hygiene [29,32] and the reasons for tooth extraction [41]. Due to the retrospective study design, a direct comparison between the study groups is not permissible. It cannot be determined whether both groups had a similar oral health status or which factors influenced the decision to perform tooth extractions. It is to be assumed that patients with tooth extractions before HNR had a worse baseline oral status, poorer mouth hygiene, and compliance regarding tooth-preserving measures. As no randomization of these factors were performed, no conclusions about causal relationships can be drawn from the results.

Moreover, tissue injuries due to tumor resection and/or neck dissection, which can overwhelmingly disturb the vasculature and mucoperiosteum of the dentoalveolar structures, were found to be an independent, modifiable factor for developing JORN [2]. Except for the information of adjuvant versus primary HNR, such data (extent of tissue injuries, especially on the jaw bone) were also poorly recorded in the hospital register and appear to be very subjective, depending upon the operator-surgeon factor (e.g., skill and experience). This aspect could explain why primary HNR did not prove to be a statistically significant risk factor for JORN in statistical analysis, despite the higher HNR dose. Furthermore, the HNR dose was identified as an independent potential risk factor in the analysis.

Concerning the general rule of thumb, the loss-to-follow-up rate in a study should be ≤20% of the sample [26]. In total, 31.8% (or 232/729) subjects were excluded from this study due to the incomplete follow-up or lack of patient consent. This is considered as an important “attrition bias” in the present research [31]. Improvements in the HNC patient register and follow-up protocol are planned, so it can be used perfectly in future investigations.

Second, previous studies highlighted that active smokers during HNR had a 32% increased risk of developing JORN [29,32]. It is understood that nicotine in cigarette smoke causes platelet aggregation and vasoconstriction, thereby increasing the risk of microvascular thromboses and poor tissue micro-perfusion. In addition, carbon monoxide competitively inhibits the binding of oxygen to hemoglobin, leading to hypoxia. These toxic substances can undermine the conditions required for wound healing, exacerbating tissue compromise and damage [29,36]. However, this study was not able to establish a significant effect of smoking on JORN. The reason for this could be that the number of cigarettes smoked per day was not collected in the radiotherapy questionnaire. For this reason, it light and heavy smokers could not be distinguished. Additionally, it was not possible to track whether smokers quit smoking during or after radiotherapy, or perhaps shortly before the survey.

Third, selection bias seems to be possible because the database was not representative of all possible patients in the population [42]. Patients may visit the family dentist or surgeon in private practice for follow-ups with/without conservative therapy for JORN and, thereby, escape recording. Selection bias (or specifically “referral bias”) would also occur if dentists or oncologists specially referred painful JORN patients to the Department of Oral and Craniomaxillofacial Plastic Surgery. This may be because they already suspected bone exposure in these patients because of the HNR history, while those with asymptomatic bone exposure may have refrained from appropriate investigations [43]. In addition, patients with a short follow-up may develop JORN at a future endpoint.

Finally, as is typical of observational studies, only association and not causation can be inferred from the results. This is because the observed association between pre-HNR TE and JORN may have been the result of confounding. In particular, it was not possible to measure and then control for, through statistical analysis, all factors that may have affected the occurrence of JORN. On the contrary, experimental studies such as clinical trials use random allocation of participants to treatment groups to control for confounding at baseline. However, the randomized design seems unethical and infeasible [41]. Nevertheless, the statistician Sir Austin Bradford Hill of the University of London proposed the criteria, which, if met, may allow causation to be inferred from an association between a risk factor and outcome in observational studies [44].

Lastly, most publications regarding the effect of pre-HNR TE and the occurrence of JORN including this study are presented in the form of an incidence report (either with or without a control group) and use different grading systems for JORN. The direct causal effect is therefore difficult to identify and requires well-designed investigations with a large cohort [3,26,29]. A comprehensive study design should account for baseline dental status, the number of decaying teeth, and the numbers of PTE and post HNR tooth extractions. In the intervention group, only teeth deemed non-restorable should be extracted, while primary focus should be tooth-preserving measures and fluoridation. Additionally, patient education and motivation to maintain optimal oral hygiene should be emphasized as integral components of the intervention strategy. Tumor location and the total dose of HNR as well as the organ doses should be considered as potential confounding factors.

## 5. Conclusions

This study addresses the relationship between PTE and JORN, which has serious implications for patient outcomes and quality of life. By employing both logistic regression and Cox regression analyses, the study explored the time dependent effects of PTE on JORN incidence. While this study was not sufficiently powered to detect small effect sizes, it was adequate to assess clinically meaningful differences in JORN risk. The findings suggest that PTE does not exert a strong protective effect against JORN and may contribute to an increased early risk, particularly within the first year post-HNR. In contrast to expectations and rationale, PTE of teeth with poor long-term prognosis before HNR does not appear to decrease the frequency of having experienced JORN to levels comparable to those in patients who did not undergo/require PTE before HNR. However, the findings of other recent studies [26,29,33,34,35,36] that pre-HNR PTE is an independent risk factor for the development of JORN could not be unreservedly confirmed, warranting the need for further research. HNR patients with PTE in this study had a 61% increased risk for JORN occurrence and were associated with 31% higher odds for experiencing JORN compared with those not requiring/receiving pre-HNR PTE, but these findings were at the best marginally significant. Although a significant relationship between the HNR dose and the occurrence of JORN was identified, no specific recommendations regarding the optimal dose for HNR can be derived, as the impact of radiation dose reduction on tumor cure rates must not be missed.

Due to the retrospective study design, a direct comparison between the study groups is not permissible, and no conclusions about causal relationships can be drawn from these results. To address the limitations of this study, the effects of oral health status, oral hygiene, smoking status, and surgery on adjacent structures, such as jaw resection and neck dissection (that could disturb blood supply to the irradiated jaw), and on the occurrence of the JORN in this cohort should be assessed. The outcome comparison between pre- versus post-HNR TE also requires further investigations. A prospective study, taking baseline dental status into account and integrating these considerations, therefore, is essential for generating reliable and actionable findings and robust evidence. Future research should also expand sample size through multi-center collaborations to improve statistical power and generalizability.

Furthermore, the radiation dose and tumor localization should be considered as potential confounding factors in the development of JORN. To clarify whether greater emphasis should be placed on tooth-preserving measures before, during, and after HNR—removing only strictly non preservable teeth prior to HNR—more robust evidence is needed. This approach could have a positive effect not only on the tumor healing chances but also on the long-term oral health of HNR patients.

## Data Availability

Deidentified individual participant data are not available. Based on the current patient data protection law in Germany, open access to the raw data is not allowed. The datasets generated and analyzed during this study are available from the corresponding author upon reasonable request.

## Appendix A

**Table A1:** Descriptive, uni- and bivariate analyses of the study cohort grouped by tooth extraction (TE).

Parameter	Overall	PTE Group	Non-PTE Group	*p*-Value (OR; 95% CI)
Study sample	497 (100)	228 (45.9)	269 (54.0)	N/A
Age	61 ± 10.95	61 ± 10.35	62 ± 11.39	*0.125* (N/A)
	(14–92)	(30–92)	(14–91)	
	Median: 61	Median: 60	Median: 62	
Male gender	382 (76.9)	177 (77.6)	205 (76.2)	*0.708* (1.084; 0.712–1.648)
*Smoking status*				
Smoker	217 (43.6)	106 (46.5)	111 (41.3)	0.198 (1.265; 0.884–1.810)
Non-smoker	272 (54.8)	117 (51.3)	155 (57.6)	
No data	8 (1.6)	5 (2.2)	3 (1.1)	
*Alcohol consumption*				
Alcohol abuse	101 (20.3)	57 (32.9)	44 (9.7)	*0.017* (*1.705*; 1.095–2.649)
No alcohol abuse	396 (79.7)	171 (75.0)	225 (84.0)	
*Comorbidities*				
Cardiovascular disease	212 (42.7)	84 (36.8)	128 (47.6)	*0.023* (*0.652*; 0.455–0.935)
Diabetes mellitus	63 (12.7)	30 (13.2)	33 (12.3)	0.788 (1.084; 0.638–1.840)
Liver disease	14 (2.8)	4 (1.75)	10 (3.71)	0.187 (0.463; 0.143–1.495)
Renal failure	24 (4.8)	14 (6.1)	10 (3.71)	0.209 (1.694; 0.738–3.891)
Respiratory disease	59 (11.9)	28 (12.3)	31 (11.5)	0.696 (1.115; 0.645–1.930)
Rheumatic disease	5 (1)	3 (1.3)	2 (0.7)	0.665 (1.780; 0.295–10.746)
*Tumor histological types*				
Squamous cell carcinoma (SCC)	456(91.8)	211 (92.5)	245 (91.1)	
NonSCC	25 (5)	13 (5.7)	12 (4.5)	
Undiff. carcinoma	4 (0.8)	1 (0.4)	3 (1.1)	
Adenocarcinoma	3 (0.6)	1 (0.4)	2 (0.7)	
Neuroendocrine tumor	3 (0.6)	0 (0)	3 (1.1)	
Adenoid cystic carcinoma	5 (1.0)	2 (0.9)	3 (1.1)	
Sarcoma	1 (0.2)	0	1 (0.4)	
*Tumor location*				0.654 (N/A)
Pharynx (tonsil, naso-, oro-, or hypopharynx)	195 (39.2)	96 (42.1)	99 (36.8)	
Oral cavity including salivary gland	203 (40.8)	93 (40.8)	110 (40.9)	
Larynx	76 (15.3)	35 (15.4)	41 (15.2)	
Nose and paranasal sinuses	23 (4.6)	4 (1.8)	19 (7.1)	
*Tumor staging*				*0.085* (1.575; 0.937–2.646)
I-II	71 (14.3)	26 (11.4)	45 (16.7)	
III-IV	425 (85.5)	202 (88.6)	223 (82.9)	
No data	1 (0.2)		1 (0.4)	
*HNR modality*				
3D-CRT	141 (28.4)	54 (23.7)	87 (32.3)	
IMRT	324 (65.2)	160 (70.2)	164 (61.0)	*0.032* (N/A)
Particle therapy	32 (6.4)	14 (6.1)	18 (6.7)	
Primary HNR (adjusted as binary versus adjuvant)	270 (54.2)	143 (62.7)	127 (47.2)	***0.0004*** **(1.909; 1.332–2.736)**
HNR dosage (Gy)	68 ± 9.3	68 ± 8.7	67 ± 9.8	0.139 (N/A)
	(30–122.4)	(30–122.4)	(30–120)	
	Median: 68	Median: 70	Median:67	
Chemotherapy	405 (81.5)	192 (85.1)	213 (79.2)	0.15 (1.402; 0.884–2.736)
ORN				
Number of cases (total)	74 (14.9)	39 (17.1)	35 (13.0)	0.209 (1.380; 0.841–2.263) ‡
Within year 1	32 (43.2)	20 (51.3)	12 (34.3)	
Within year 5	71 (95.9)	36(92.3)	35(100)	
Later than year 5	3 (4)	3 (7.7)	0 (0)	
Location				
Mandible	67 (9.5)	36 (92.3)	31 (88.6)	
Maxilla	5 (6.8)	1 (2.6)	4 (11.4)	
Both maxilla and mandible	2 (2.7)	2 (1.3)	0 (0)	
Interval between TE and ORN (days)	753 ± 742	724 ± 727	786 ± 769	0.721 (N/A)
	(37–3861; 74)	(37–3100; 39)	(70–3861; 35)	
	Median: 465	Median: 359	Median: 557	
Follow-up (days; number of cases)	1531 ± 1290	1359 ± 1310	1677 ± 1258	*0.006* (N/A)
	(91–6287; 497)	(91–5347; 228)	(92–6287; 269)	
	Median: 1161	Median:859	Median: 1451	

Note: Continuous measurements are listed as mean ± standard deviation, range in (); categorical measurements are listed as number, percentage in (). OR—odds ratio; CI—confidence interval; N/A—not available. Statistically significant *p* values are indicated in bold typeface. Almost statistically significant *p* values (*≤0.15* but *>0.005*) are indicated in italic typeface; ‡ Number needed to harm (NNH) = 25.

## Appendix B

**Table A2:** Abbreviations.

Abbreviation	Explanation
3D-RT	3D Conformal Radiotherapy
HNC	Head and Neck Cancer
HNR	Head and Neck Radiotherapy
HR	Hazard Ratio (Statistical term)
IMRT	Intensity-Modulated Radiotherapy
JORN	Jaw Osteoradionecrosis
MSK	US Memorial Sloan Kettering Cancer Center
NNH	Number Needed to Harm (statistical term)
OR	Odds Ratio (statistical term)
PHR	Proportional Hazard Ratio (statistical term)
POR	Prevalence Odds Ratio (statistical term)
PTE	Prophylactic Tooth Extraction
SRMA	Systematic Reviews with Meta-Analysis
TE	Tooth Extraction
TNM Stage	Tumor-Node-Metastasis Stage (tumor classification)
UKGM	University Hospital Gießen and Marburg
